# Common shared genetic variation behind decreased risk of breast cancer in celiac disease

**DOI:** 10.1038/s41598-017-06287-9

**Published:** 2017-07-19

**Authors:** Emilio Ugalde-Morales, Jingmei Li, Keith Humphreys, Jonas F. Ludvigsson, Haomin Yang, Per Hall, Kamila Czene

**Affiliations:** 1 Department of Medical Epidemiology and Biostatistics, Karolinska Institutet, Stockholm, Sweden; 2 Human Genetics, Genome Institute of Singapore, Singapore, 138672 Singapore; 3 Department of Pediatrics, Örebro University Hospital, Örebro, Sweden

## Abstract

There is epidemiologic evidence showing that women with celiac disease have reduced risk of later developing breast cancer, however, the etiology of this association is unclear. Here, we assess the extent of genetic overlap between the two diseases. Through analyses of summary statistics on densely genotyped immunogenic regions, we show a significant genetic correlation (*r* = −0.17, s.e. 0.05, *P* < 0.001) and overlap (*P*_permuted_ < 0.001) between celiac disease and breast cancer. Using individual-level genotype data from a Swedish cohort, we find higher genetic susceptibility to celiac disease summarized by polygenic risk scores to be associated with lower breast cancer risk (OR_per-SD_, 0.94, 95% CI 0.91 to 0.98). Common single nucleotide polymorphisms between the two diseases, with low *P*-values (*P*_CD_ < 1.00E-05, *P*_BC_ ≤ 0.05), mapped onto genes enriched for immunoregulatory and apoptotic processes. Our results suggest that the link between breast cancer and celiac disease is due to a shared polygenic variation of immune related regions, uncovering pathways which might be important for their development.

## Introduction

Breast cancer risk has been reported to be consistently lower among celiac disease patients, ranging from being 10-15% lower in Nordic studies^1–4^ to as much as 50-80% lower in other European studies with smaller sample sizes^5–8^. Celiac disease is a lifelong gastrointestinal disease characterized by villous atrophy and inflammation in the small intestine^9^. It occurs in about 1% of the Caucasian population and is triggered by gluten exposure^10^. There is little in literature to clarify why a diagnosis of celiac disease confers protection against breast cancer. Explanations that have been forwarded include a lower body mass index and lower estrog﻿en exposure bo﻿th ﻿as a consequence of celiac disease; for example, characteristics secondary to undernutrition such as later menarche and earlier menopause commonly observed at high frequencies among women with celiac disease are associated with decreased risk of breast cancer^2^. A third hypothesis involves the role of immunogenic factors in breast cancer development and progression^11^. Inverse relationships have also been observed between breast cancer and other inflammatory disorders such as ulcerative colitis and rheumatoid arthritis, which is consistent with a possible involvement of the immune system in the etiological pathway of breast cancer^2^. Others have also shown that the interplay between hormonal and immune-related mechanisms can shape mammary tissue development^12^.

Both breast cancer and celiac disease have strong genetic components. Heritability is estimated to be between 25 to 31%^13, 14^ and 68 to 75%^15^ for breast cancer and celiac disease, respectively. Heritability of complex diseases such as celiac disease and breast cancer is highly polygenic, which means that it is controlled not just by one gene, but rather, by multiple genes^16^. For example, genome-wide association studies (GWAS) interrogating upwards of ~200,000 single nucleotide polymorphisms (SNPs) have identified ~40 loci associated with celiac disease^17^, and more than twice as many (107 loci) for breast cancer^18^. Studies performed on different phenotypes have shown that certain genetic loci can be associated with seemingly distinct traits, otherwise known as pleiotropy^19^. It is also known that many diseases and traits exhibit significant coheritability^20^ and have shared genetic components^21^, and such genetic associations often reveal clues about novel mechanisms and pathways.

With the emergence of large-scale GWAS studies, it is timely to leverage on the data collected from international consortia to investigate possible causal trait relationships^22^ using clinical and epidemiological data as a guide^23–25^. Motivated by evidence of an association between celiac disease and breast cancer from epidemiologic studies, the aims of this study are to test whether these findings could be due to shared genetic determinants, and to elucidate what mechanisms are potentially responsible for the common connection.

## Results

### Inverse genetic correlation and genetic overlap between breast cancer and celiac disease

Genetic correlation is indicative of shared genetic etiology. It refers to common genetic variation associated with a pair of phenotypic traits, assuming an additive model. Given the epidemiological association between breast cancer and celiac disease, as well as their strong genetic heritabilities, we used GWAS summary statistics for each disease to estimate genetic correlation. For breast cancer, data was obtained from the Collaborative Oncological Gene-Environment Study (COGS) consortia (http://www.cogseu.org/). The study includes individuals of European ancestry genotyped on a custom Illumina iSelect Array (iCOGS)^26^, which comprises 211,155 SNPs. iCOGS was designed to understand genetic susceptibility of three hormone related cancers: breast, ovarian, and prostate. As breast cancer is a heterogeneous disease, we also included two major subtypes of breast cancer based on estrogen receptor (ER) status. In the COGS, analyses had been conducted on 46,785 breast cancer cases and 42,892 controls to estimate breast cancer risk overall, 27,078 ER-positive cases and 42,111 controls for ER-positive breast cancer risk, and 7,333 ER-negative cases and 42,468 controls for ER-negative breast cancer risk^18^. Summary statistics for celiac disease (133,352 SNPs) were downloaded from the ImmunoBase (https://www.immunobase.org/), a web based resource focused on the genetics and genomics of immunologically related human diseases. Celiac disease data have been reported in a GWAS study by Trynka *et al*.^17^ on 12,041 celiac disease cases and 12,228 controls of European ancestry using the Illumina Infinitum High-Density array (ImmunoChip), interrogating 195,806 SNPs and designed to target immune associated genome regions^27^. To improve the comparability between the two datasets, we used summary statistics for 173,301 SNPs in the iCOGS study imputed against the 1000 Genomes Project (1KG) March 2012 release reference panel^18^, which were also present on the ImmunoChip. The datasets were matched based on chromosome and SNP base pair positions, which resulted in 129,618 celiac disease SNPs used as input.

**Figure 1:**
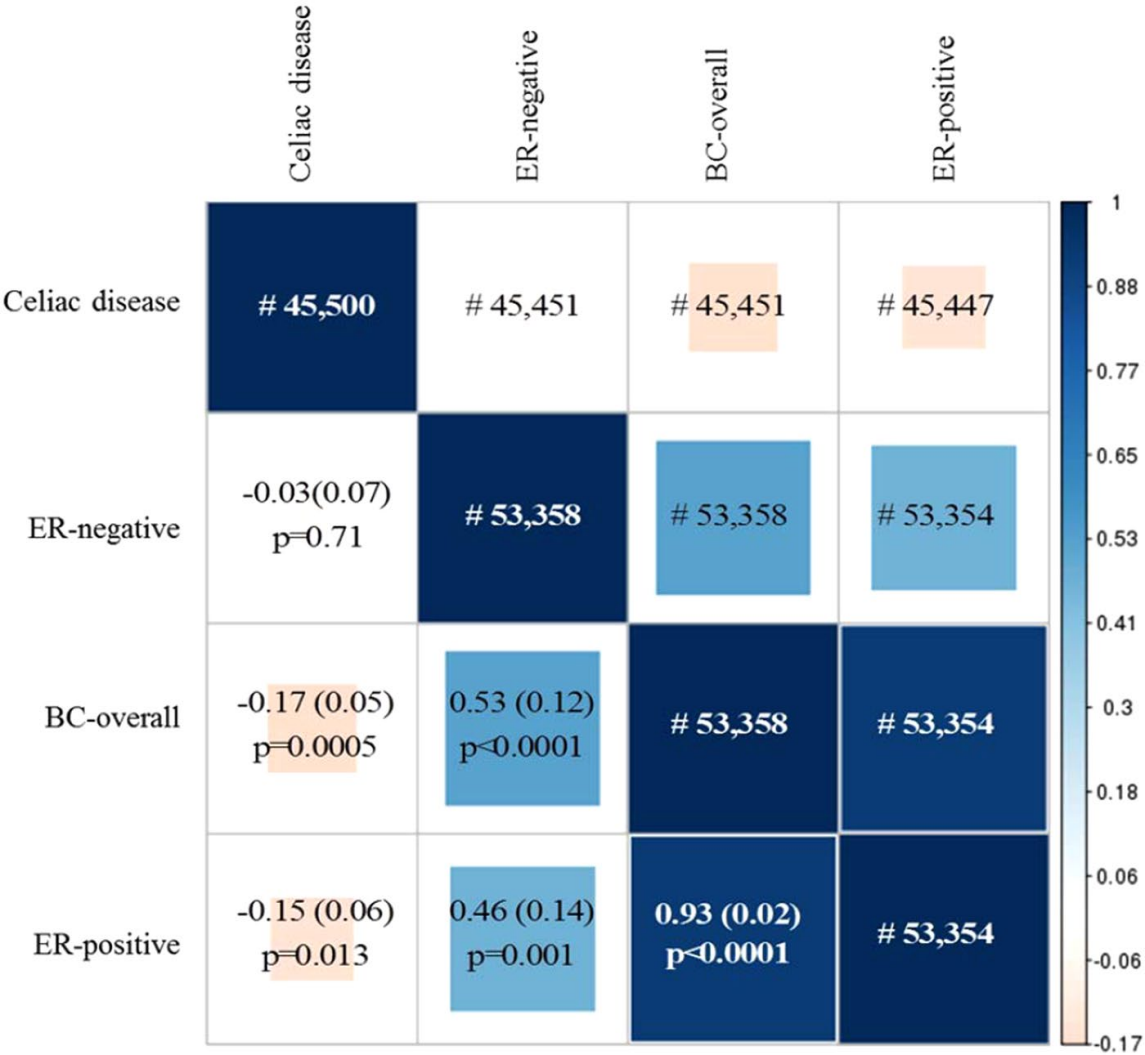
Genetic correlation using LD score regression. Plot is based on LDSC regression coefficients for each comparison pair (e.g. BC vs CD, BC_ER-negative_ Vs CD, BC_ER-positive_ Vs CD, etc.). Subdiagonal cells indicate the respective correlation coefficient and (standard error). Square size and color are scaled according to the correlation coefficient *r*. Traits are paired using hierarchical cluster analysis. ^#^Number of overlapping SNPs included in the LDSC regression.

Genetic correlation between breast cancer and celiac disease was analyzed using LD score regression (LDSC)^28^ to model effect size estimates for immunogenic SNPs in both diseases. Therefore, rather than studying cross-correlation, we are studying the role of celiac disease risk loci in breast cancer susceptibility. Given the observed reduced risk of breast cancer in celiac disease patients, we would expect an inverse genetic correlation. After LDSC filtering procedure and merge with reference LD scores (Supplementary Table 1), genetic correlation analyses included 45,451, 45,451 and 45,447 matching SNPs between the celiac disease and breast cancer overall, ER-positive and ER-negative datasets, respectively. Significant inverse genetic correlations (*r*) were found between celiac disease and overall breast cancer (*r* = −0.17, s.e. 0.05, *P* = 0.0005) and ER-positive breast cancer (*r* = −0.15, s.e. 0.06, *P* = 0.01), but not for ER-negative breast cancer (*r* = −0.03, s.e. 0.07, *P* = 0.71) (Fig. 1).

Further interrogation of shared common genetic components between the two diseases was carried out using SNP effect concordant analysis (SECA)^29^, where SNP effect size estimates were tested for concordant or discordant effects, analogous to genetic correlation tested with LDSC. Additionally, SECA also assesses the extent of genetic overlap (enrichment of overlapping SNPs between the two traits with low P-values). For each dataset pair comparison, SECA aligned and selected 15,365, 15,400, 15,428 independent SNPs that are common between the celiac disease and overall, ER-positive, and ER-negative breast cancer datasets, respectively (Supplementary Table 2). In the primary analyses which are summarized in Supplementary Figure 1, genetic overlap (defined as excess of celiac disease SNPs in overlap with breast cancer datasets) was significant between celiac disease and breast cancer overall (*P*_BT-permuted_ = <0.001), ER-positive (*P*_BT-permuted_ < 0.001), and ER-negative (*P*_BT-permuted_ < 0.001). SNP effect discordance (inverse correlation) between celiac disease and breast cancer overall (*P*_FT-permuted_ = 0.059), ER-positive (*P*_FT-permuted_ = 1.000), and ER-negative (*P*_FT-permuted_ = 0.278) did not reach significance. In order to determine size of the association, SECA identified a subset of overlapping SNPs yielding the most significant correlation, namely, minimum concordance or discordance. This analysis was carried out studying the association between celiac disease and breast cancer overall (OR_FT-min_, 0.60, 95% CI 0.44–0.82, *P*_FT-min_ = 0.001), ER-positive (OR_FT-min_, OR, 0.86, 95%CI, 0.74–1.00 *P*_FT-min_ = 0.05), and ER-negative (OR_FT-min_, OR, 0.73, 95%CI, 0.57–0.95 *P*_FT-min_ = 0.02) (Table 1). After adjusting for multiple testing, only the association for overall breast cancer (which showed discordance) remained significant (*P*_FTmin-permuted_ = 0.022).

**Table 1:** Inverse genetic correlation for the SNPs subset yielding most significant association (minimum discordance).

Celiac disease and breast cancer:	OR_FT-min_ (95% CI)	*P*_FT-min_	*P*_FT-min permuted_
Overall	0.60 (0.44 to 0.82)	0.001	0.022
ER-positive	0.86 (0.74 to 1.00)	0.050	0.319
ER-negative	0.73 (0.57 to 0.95)	0.019	0.187

Genetic correlation estimates by SECA Fisher’s tests (FT) identifying minimum discordance (FT-min) in subsets of overlapping SNPs between breast cancer and celiac disease. OR and its CI range is presented for the SNP subsets yielding minimum discordance, which refers to the SNP subset with the lowest Fisher’s P-value (*P*_FT-min_). P-value was adjusted for multiple testing by a permutation procedure (*P*_FT-min permuted_).

### Higher celiac disease genetic susceptibility associated with decreased breast cancer risk

**Figure 2:**
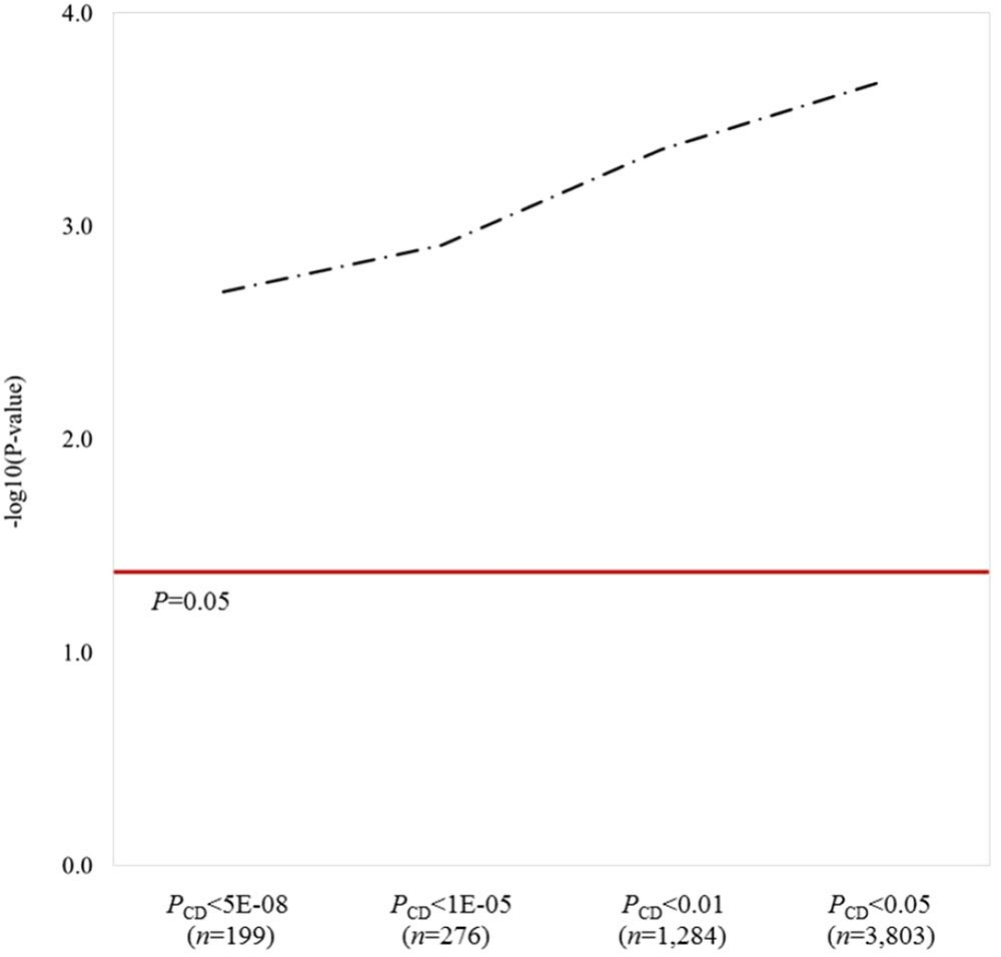
Significance of celiac-PRS profiles for association with overall breast cancer risk. Profiles are based on CD-SNPs under given *P*_CD_ thresholds. P-values are transformed into negative logarithmic scale base 10. Red solid line denotes threshold for a nominally significant association (*P* < 0.05).

In a third approach, we tested whether top SNPs for celiac disease (CD-SNPs) could predict breast cancer status in a group of women. Genetic susceptibility to celiac disease was summarized using polygenic risk scores (celiac-PRS). In essence, celiac-PRS accounts for the genetic susceptibility of an individual based on the risk allele load weighted by the SNP effect sizes reported by the celiac disease GWAS, under an additive model. Celiac-PRS was analyzed as an exposure variable in a case-control study comprised of 5,002 breast cancer cases and 5,433 controls from the pKARMA cohort. Celiac-PRS was found to be inversely associated with overall and ER-positive breast cancer risk in a dose-dependent manner (*P*-trend < 0.02) (Table 2). Celiac-PRS based on 199 genome-wide significant CD-SNPs was associated with 6% lower risk of overall (OR_per-SD_, 0.94, 95% CI 0.91 to 0.98, *P* = 0.002) and ER-positive breast cancer (OR_per-SD_, 0.94, 95% CI 0.90 to 0.98, *P* = 0.004), and 2% for ER-negative breast cancer (OR_per-SD_, 0.98, 95% CI 0.90 to 1.06, *P* = 0.54). The risk was 13% lower in individuals with the highest genetic susceptibility to celiac disease (4^th^ celiac-PRS quartile compared to 1^st^ quartile) for both overall and ER-positive breast cancer risk (overall: OR_Q4_, 0.87, 95% CI 0.78 to 0.97, *P* = 0.016; ER-positive: OR_Q4,_ 0.87, 95% CI 0.77 to 0.98, *P* = 0.022). The risk was up to 17% lower in women with highest susceptibility when the celiac-PRS included 3,803 SNPs nominally associated with celiac disease (i.e. *P*_CD_ < 0.05) (overall: OR_Q4_, 0.83, 95% CI 0.75 to 0.93, *P* = 0.001; ER-positive: OR_Q4_, 0.83, 95% CI 0.74 to 0.93, *P* = 0.002). As expected under a polygenic model, including more CD-SNPs in the profiles improved the strength of the association with breast cancer risk (yielding smaller *P*-values) (Fig. 2). In a case-only study, no significant difference was observed within tumor subgroups defined by ER-status, lymph node involvement status, HER2 status, tumor grade, and tumor size (Supplementary Table 3).

**Table 2:** Association of celiac-PRS profiles with breast cancer risk.

Profiles (quartile range)	BC-Overall				ER-positive (*n* = 3,804)				ER-negative (*n* = 695)			
	OR	95%CI	*P*-value	*P*-trend	OR	95%CI	*P*-value	*P*-trend	OR	95%CI	*P*-value	*P*-trend
**GWAS significant (** ***P***_**CD**_ < **5E-08)** – 199 SNPs												
Q1 (−0.0992 to −0.065)	1.00	Reference			1.00	Reference			1.00	Reference		
Q2 (−0.065 to −0.0554)	1.02	0.92 to 1.14	0.716		0.99	0.89 to 1.12	0.928		1.16	0.93 to 1.45	0.190	
Q3 (−0.0554 to −0.0366)	0.99	0.89 to 1.10	0.809		0.96	0.86 to 1.08	0.519		1.14	0.91 to 1.42	0.264	
Q4 (−0.0366 to 0.0694)	0.87	0.78 to 0.97	0.016	0.015	0.87	0.77 to 0.98	0.022	0.020	0.96	0.76 to 1.21	0.715	0.693
Continuous variable	0.94	0.91 to 0.98	0.002		0.94	0.90 to 0.98	0.004		0.98	0.90 to 1.06	0.540	
***P***_**CD**_ < **1.00E-05** – 276 SNPs												
Q1 (−0.0717 to −0.0469)	1.00	Reference			1.00	Reference			1.00	Reference		
Q2 (−0.0469 to −0.0401)	0.98	0.88 to 1.09	0.694		0.95	0.84 to 1.06	0.352		1.12	0.90 to 1.40	0.317	
Q3 (−0.0401 to −0.0263)	0.97	0.87 to 1.08	0.632		0.96	0.86 to 1.08	0.514		1.07	0.86 to 1.34	0.542	
Q4 (−0.0263 to 0.0505)	0.85	0.77 to 0.95	0.005	0.008	0.85	0.76 to 0.96	0.008	0.015	0.91	0.72 to 1.15	0.434	0.399
Continuous variable	0.94	0.90 to 0.98	0.001		0.94	0.90 to 0.98	0.002		0.97	0.90 to 1.05	0.499	
***P***_**CD**_ < **0.01** – 1,284 SNPs												
Q1 (−0.0151 to −0.01)	1.00	Reference			1.00	Reference			1.00	Reference		
Q2 (−0.01 to −0.00849)	0.91	0.82 to 1.02	0.097		0.90	0.80 to 1.01	0.064		0.95	0.76 to 1.18	0.622	
Q3 (−0.00849 to −0.00537)	0.97	0.87 to 1.08	0.545		0.95	0.85 to 1.07	0.419		1.03	0.83 to 1.28	0.765	
Q4 (−0.00537 to 0.0116)	0.81	0.72 to 0.90	0.0001	0.001	0.80	0.71 to 0.90	0.0002	0.002	0.84	0.66 to 1.05	0.123	0.239
Continuous variable	0.93	0.90 to 0.97	0.0004		0.93	0.89 to 0.97	0.001		0.97	0.89 to 1.05	0.428	
***P***_**CD**_ < **0.05** – 3,803 SNPs												
Q1 (−0.00522 to −0.0035)	1.00	Reference			1.00	Reference			1.00	Reference		
Q2 (−0.0035 to −0.00295)	0.96	0.87 to 1.07	0.504		0.94	0.83 to 1.05	0.268		1.00	0.80 to 1.24	0.965	
Q3 (−0.00295 to −0.00191)	0.94	0.84 to 1.05	0.261		0.91	0.81 to 1.02	0.114		1.09	0.88 to 1.36	0.429	
Q4 (−0.00191 to 0.00393)	0.83	0.75 to 0.93	0.001	0.001	0.83	0.74 to 0.93	0.002	0.002	0.88	0.70 to 1.11	0.277	0.465
Continuous variable	0.93	0.89 to 0.97	0.0002		0.93	0.89 to 0.97	0.0003		0.97	0.89 to 1.05	0.457	

Breast cancer risk association with celiac-PRS profiles including CD-SNPs with P-value less than four significance thresholds [*P*_CD_ < 5E-08, *P*_CD_ < 1E-05, *P*_CD_ < 0.01, and *P*_CD_ < 0.05]. Celiac-PRS quartiles (Q1–Q4) were defined based on PRS distribution in controls. Celiac-PRSs as continuous variables expressed per 1 standard deviation.

### Candidate immune response genes and pathways underlying the association between celiac disease and breast cancer

To identify celiac disease genes and molecular pathways involved in breast cancer susceptibility, we performed enrichment analysis using Data-driven Expression Prioritized Integration for Complex Traits (DEPICT)^30^. The method assumes that the selected loci surpass the genome-wide significant threshold (*P* < 5.00E-08). However, since genome-wide significant variants did not determine the genetic overlap between breast cancer and celiac disease, we changed the threshold to include variants with moderate signals. From the 15,365 independent SNPs defined in the SECA analysis, we selected SNPs which had suggestive association with celiac disease (118 SNPs under *P*_CD_ < 1.00E-05). Given that the genetic overlap was limited to immune-related genomic regions genotyped for celiac disease, we ranked overlapping SNPs by their evidence of association (by P-values) relative to the overlap with breast cancer to reduce the possibility that the prioritized genes could be due to chance (sensitivity analysis). SNPs were divided into two SNP subsets, *P*_BC_ ≤ 0.05 and *P*_BC_ > 0.05, containing 52 and 66 SNPs respectively. If the loci were truly important for the genetic association between celiac disease and breast cancer, findings based on second SNP subset would be less reliable. Hits derived from SNPs not nominally associated with breast cancer were therefore removed from the initial findings. The SNPs with smallest breast cancer P-value (*P*_BC_ < 0.0001) were rs114762590 and rs115258774, both of which were significantly associated with celiac disease surpassing the genome-wide significant threshold and had opposite direction to breast cancer. Summary statistics for the top 52 SNPs are shown in Supplementary Table 4.

Genes mapping onto the 52 ‘top’ overlapping SNPs were significantly overrepresented in 21 biological processes (FDR < 0.05). After the sensitivity analysis (yielding 600 significant processes at FDR < 0.01), 14 processes remained as unique hits and were mainly related to induction of programmed cell death, MAP3K7 cytokine-activated transduction pathway, other signaling protein-protein interaction subnetworks, myeloid leukocyte differentiation, as well as decreased cell number of leukocytes and lymphocytes (Supplementary Table 5). To identify genes most likely underlying these biological processes, DEPICT identified 15 prioritized genes that were deemed most relevant based on their probability to enrich for the same biological processes as other candidate genes (i.e. redundant genes were removed, FDR < 0.05) (Table 3). We found 13/15 genes to be exclusively prioritized from the analysis of 52 ‘top’ SNPs associated with celiac disease and overlapping with breast cancer at *P*_BC_ ≤ 0.05 (Supplementary Figure 2).

**Table 3:** DEPICT prioritized genes mapping onto 52 ‘top’ overlapping SNPs.

Locus	Genes in locus (*n*)	Chromosome and position	Gene symbol	*P*-value	Closest to lead SNP
rs2755244	3	chr1:67278568-67519782	MIER1*	1.87E-07	FALSE
rs4886410	15	chr15:74701630-75230509	CSK*	1.34E-06	TRUE
rs4886410	15	chr15:74701630-75230509	SCAMP2*	1.36E-05	FALSE
rs76830965	2	chr3:159631189-159943086	IL12A*	2.57E-05	TRUE
rs4886410	15	chr15:74701630-75230509	SEMA7A	0.00067	FALSE
rs78756788	5	chr2:102803433-103327777	IL18RAP	0.0011	TRUE
rs78756788	5	chr2:102803433-103327777	IL18R1^**§**^	0.0012	FALSE
rs11903660	1	chr2:204732509-204738683	CTLA4^**§**^	0.0013	TRUE
rs9347286;rs79715597	2	chr6:159397312-159466184	TAGAP	0.0021	TRUE
rs225132	3	chr1:7979907-8086368	TNFRSF9	0.0022	FALSE
rs115102354	2	chr3:46205096-46308197	CCR1	0.0022	FALSE
rs2755244	3	chr1:67278568-67519782	SLC35D1	0.0025	TRUE
rs11680095	1	chr2:182321619-182400914	ITGA4	0.003	TRUE
rs864537	1	chr1:167399877-167487847	CD247	0.004	TRUE
rs11847049	1	chr14:69254377-69263190	ZFP36L1	0.006	TRUE

Genes with FDR adjusted *P*-value lower than 0.05. *FDR < 0.01; ^**§**^hits also found significant (FDR < 0.01) in the sensitivity analysis (66 CD-SNPs, *P*_BC_ > 0.05) were considered as unreliable findings. ^§^FDR < 0.01.

## Discussion

In agreement with epidemiological studies showing lower breast cancer risk in celiac disease patients, we found an inverse genetic association between the two diseases using three different methods. There was no evidence that the association between genetic susceptibility to celiac disease and lower risk of breast cancer differed by tumor characteristics. We also prioritized apoptotic and immune-related genes that could represent important etiological factors underlying the reduced risk of breast cancer in celiac disease.

In spite of the strong heritability of breast cancer and celiac disease, the etiological role played by their genetic components remains to be uncovered. Both prospective cohort studies^31–33^ and randomized clinical trials^34, 35^ have found that breastfeeding duration and age at gluten introduction may be less important than previously thought for the etiology of celiac disease. Instead, it seems that genetic factors^15, 36^ determine the risk of celiac disease. For breast cancer, immune response factors have been shown to be important factors associated with prognosis^37^, and potentially to breast cancer susceptibility^38^. As for genetic markers of breast cancer, studies on immune response candidate genes have identified few single risk alleles for specific populations^39, 40^.

In our study, inverse genetic correlation between breast cancer and celiac disease were consistent across different methods, indicating that the link between the two diseases is a result of shared immunogenetic components. Through LDSC and SECA methods which use all SNPs with available summary statistics for pair of traits, we found significant genetic correlation and overlap between breast cancer and celiac disease. While LDSC uses all common SNPs between the two diseases to estimate genetic correlation (r = −0.17), SECA identified inverse correlation in the most significant subset of SNPs (OR = 0.60), indicating the presence of allelic effects that increases risk for one disease and decreases risk for the other. The fact that genetic discordance (negative correlation) was not found significant in the SECA primary analyses as compared to the LD score regression, could be due to loss of power and related to the different approaches they use to deal with LD structure (see methods). In a third approach, genetic correlation was estimated by first summarizing the per-individual allelic load using a polygenic risk score, and then regressing the effect in a case control set up. We found 6 up to 17% lower risk to breast cancer to be comparable to the 10–15% decreased risk reported in Nordic epidemiological studies^1–4^. Given shared environmental exposures which could mediate the association, we considered GWAS summary statistics data for body mass index (BMI) from the GIANT consortium^41^. The average BMI has been shown to be lower in celiac disease patients, while low BMI is also associated with lower risk of breast cancer. However, we did not find any indication of a genetic correlation between BMI and breast cancer or celiac disease (*P* = 0.23 and *P* = 0.79 respectively, data not shown).

The involvement of the immune system is typically associated with ER-negative disease. Lymphocytic infiltration has been reported as a favorable prognostic factor for ER-negative^42^ and triple-negative breast cancer^43^. ER-negative breast cancer is characterized by a stronger immunogenic component, which could be proposed as the underlying link with celiac disease. Still, we did not observe genetic correlation between celiac disease and ER-negative breast cancer, most probably due to the low statistical power as a consequence of a smaller sample size for the ER-negative datasets.

Since complex traits such as breast cancer and celiac disease involve the deregulation of multiple interrelated biological processes, it may be informative to isolate which genes are important in the etilology of both diseases. In our functional enrichment results, we found genes involved in relevant mechanisms to be implicated in developmental and immunoregulatory processes. The most significantly prioritized genes were: *Mesoderm Induction Early Response 1* (*MIER1*), *C-src Tyrosine Kinase* (*CSK*), *Secretory Carrier Membrane Protein 2* (*SCAMP2*), and *Interleukin 12 Alpha* (*IL12A*)*. MIER1* codes for proteins with transcriptional repressive function and has been found upregulated in human breast carcinoma cell lines and tumors^44^. By interaction with transcription factors and chromatin modifiers such as ER-alpha and histone deacetylase inhibitor HDAC1/2^45, 46^, *MIER1-alpha* inhibits estrogen dependent growth and lack nucleus internalization during breast cancer progression^47^. Thus, is possible that genetic variants could affect *MIEK1* protein interactions and migration mechanisms necessary to exert its function, explaining the high expression level seen in breast cancer cells as a compensatory response. *CSK* gene codes for the human cytosolic non-receptor tyrosine kinase protein, which regulate different transduction signals implicated in cell growth, differentiation, migration and immune response processes. CSK inactivates the sarcoma (Src) family kinases which otherwise would lead to T-Cell antigen specific response by phosphorylating zeta chain T-cell receptor (TCR), which has been found downregulated for different cancer types, autoimmune disease and chronic inflammation^48^. *IL12A* codes for a cytokine with important effects on the regulation of immune and inflammatory responses^49^ and has been considered for cancer immunotherapy^50^ *. ILI12A* loci selected through genetic population factors have been associated with celiac disease and other autoimmune diseases^51^. A query in a pathway catalog (http://pathcards.genecards.org; accessed on November 15, 2016) showed that other significantly prioritized genes (*SEMA7A*, *ITGA4, IL18RAP*, *CD24*, *TAGA*, *TNFRSF*, *ZFP36L1*) are classified on immune-related signaling pathways with important immunomodulatory^52, 53^ and apoptotic functions^54^. Overall, this suggests that a complex network of signaling pathways play an important role in the regulation of the immune response and surveillance. Disruption of this network could lead to autoimmune responses, or to changes in mammary microenvironment elements predisposing to cancer immune evasion, in line with cumulative evidence highlighting the relevance of host immunity and genomic alterations in the disease heterogeneity and for tailoring therapeutic interventions^55^. It could be hypothesized that while some of the overlapping variants might be involved in heightened immune responses, they could at the same time increase immunosurveillance against carcinogenic processes in breast tissue, thereby reducing breast cancer risk. Our findings might guide future studies that can help to understand the role played by the immune system in breast cancer susceptibility.

The main strength of our study is the use of reliable summary statistics from large multicenter GWAS consortia for both diseases, and the leverage of epidemiological association to estimate genetic correlation and immune-related genetic susceptibility to breast cancer. By using different polygenic approaches and prior biological knowledge, we could detect novel associations. It is notable that the shared genetic component between celiac disease and breast cancer was not driven by strong signals (e.g. SNPs surpassing stringent GWAS threshold), but rather determined by several weaker signals, namely ‘suggestive variants’. Although this type of variants are typically not the ones identified in conventional genome-wide or candidate gene association studies, they may still be indicative of biological importance. A notable limitation is that the custom SNP chips used by the respective consortia target different regions of the genome, which reduces comparability, even when imputation of breast cancer genotypes is used. We also explored the use of methods such as Direct Imputation of Summary Statistics (DIST)^56^ on celiac disease dataset, which increased the number of common variants with good quality imputation (INFO > 0.9), to approximately 500 K. However, the use of this method did not improve the comparability between the two diseases. After LD-based pruning as performed in SECA analysis, ~14 K independent SNP remained for comparison. Despite its restrictions, the ImmunoChip provides information on the most important genetic components of celiac disease (mainly at both HLA and no-HLA regions) and therefore can be used to highlight an otherwise undermined immunogenic role in breast cancer susceptibility. Our analysis should not be regarded as a full genome assessment of the genetic overlap between the two diseases, but rather as an assessment of the shared genetic variation of immune-related regions. If we had had access to celiac disease individual-level genotype data, genetic correlation analysis using other robust methods such as the GCTA–GREML^57^ would have been possible. It is however unclear whether imputation based on raw data could allow for a more comprehensive comparison of the genetic variation between the two diseases. It is also possible that deeper genome coverage could improve the assessment of the genetic overlap and facilitate the identification of common causal variants.

In summary, we show evidence of a shared genetic component underlying the link between the two diseases at immune-related regions. The protective effect associated to higher load of celiac disease genetic susceptibility, summarized by the celiac polygenic risk score in a Swedish cohort, suggest that a less responsive immune system is implicated in the predisposition to breast cancer. While considering that our analyses were constrained by the immune-related genomic coverage, we used functional annotation analyses to identify genetic loci known to be involved in the complex regulation of the innate immune response which are likely to underlie common etiological basis between the two diseases. Replication of our findings and refined analysis related to disease subtypes will require larger samples sizes and better genotype data. Functional analyses integrating other layers of Omics data will be helpful to identify and validate specific mechanisms underlying breast cancer development, and possibly shed light on breast cancer prevention and treatment strategies.

## Methods

### Genetic correlation and overlap tests using GWAS summary statistics

Genetic correlation was estimated using the cross-trait LD score regression (LDSC)^28^ software (v1.0.0) on matching SNPs surpassing LDSC filter procedure. Given that imputation quality correlates with LD score, HapMap3 SNPs with European MAF > 1% (w_hm3.snplist) were filtered with the -merge-allele flag. LDSC defines genetic correlation between a pair of traits as the genetic covariance normalized by heritabilities on each phenotype accounted by the genotyped variants (SNPs) across the genome. Genetic covariance is estimated under a model where standardized genotype effects sizes are treated as random and is, in practice, estimated by regressing z-scores on sample size weighted linkage disequilibrium (LD) scores. In our analyses, we only included SNPs with reliable LD scores available in the in-software file (w_hm3.snplist), comprised of 1,217,311 SNPs with pre-computed LD scores estimated from European-ancestry samples in the 1 KG reference panel (see online Methods in Bulik-Sullivan *et al*.^58^), using the ‘merge-alleles’ flag.

SNP Effect Concordance Analysis (SECA)^29^ was used to test genetic overlap and SNP effect direction (analogous to genetic correlation tested by LDSC above). For each pair of datasets (celiac disease against overall, ER-positive, and ER-negative breast cancer), matching SNP were selected through SECA filtering and alignment procedures. Independent (index) SNPs were selected by SECA through a two-step ‘*P*_BC_-value informed” LD-clumping procedure (first round: pairwise LD r^2^ > 0.1 within 1 Mb windows; second round: pairwise LD r^2^ > 0.1 within 10 Mb windows) based on 1 KG v3 CEU (b37 rsIDs; MAF > 1%). Following SECA scripts, pleiotropy tests were performed for each dataset pair on 144 subsets defined by combinations of 12 × 12 P-value thresholds on breast cancer (*P*_BC_) and celiac datasets (*P*_CD_), that is {*P*_BC_, *P*_CD_} = {0.01, 0.05, 0.1, 0.2, 0.3, 0.4, 0.5, 0.6, 0.7, 0.8, 0.9, 1.0}. Genetic overlap was analyzed using binomial tests (BT) to determine whether there is an excess [observed (obs) ≥ expected (exp)] of SNPs with overlapping p-values (obs). Because GWAS are expected to produce an excess of lower P-values, the ‘overlap’ null probability (e.g.’expected proportion’) is defined as the observed proportion of celiac disease SNPs under the given *P*_CD_. Genetic concordance was analyzed using Fisher’s tests (FT) performed on 2 × 2 contingency tables for the SNP effect direction (positive or negative) on both datasets. The per-subset SNP effect direction is defined as concordant (positive correlation) when there is a significant larger proportion of SNPs with the same direction in both datasets (FT’s OR > 1; *P* < 0.05), and discordant (negative correlation) when in the opposite direction (FT’s OR < 1; *P* < 0.05). Primary tests were performed via permutation by repeating the analyses of the 144 subsets on one thousand uncorrelated datasets generated by randomly shuffling the observed SNP effect (BETA) and corresponding P-value between SNPs in breast cancer datasets. Empirical (permuted) P-values indicate whether the observed number of subsets with significant overlap (*P*_BT_ < 0.05) or concordance/discordance (*P*_FT_ < 0.05) are more than expected by chance (*P*_permuted_ < 0.05). Minimum discordance was identified on SNP subsets yielding the lowest FT’s P-value, and adjusted for multiple testing (*P*_min-permuted_).

LDSC and SECA differ in their approaches to evaluating whether pairs of phenotypes have a shared genetic basis. LDSC is model based and treats genotype effects as random, whilst SECA is based on a fixed effects approach. The approaches deal in different ways with the issue that SNPs in high LD with an unknown causal variant will represent an important inflating factor^59^. LDSC deals with this using an LD-score weighting procedure, whilst SECA applies a strict LD pruning procedure to select a set of unbiased index SNPs. As a result, fewer SNPs were included in SECA analyses (~15,300) than in LDSC regressions (~45,500).

### Association of celiac-PRS with breast cancer risk

We constructed celiac disease PRS profiles (celiac-PRS) using individual level genotype data for subjects in the pKARMA study genotyped as part of the iCOGS initiative. pKARMA is made up of 5,002 invasive breast cancer cases (from the Linne-Brost 1 (Libro1) study) and 5,433 controls (from the Karolinska Mammography Project for Risk Prediction of Breast Cancer (KARMA^60^)). Libro1 consists of female primary breast cancer cases diagnosed in Stockholm between January 2001 and December 2008 identified via the Regional Cancer register^61^. Tumor characteristics were retrieved from the Stockholm-Gotland Regional Breast Cancer quality registry^62^. ER status was recorded as negative or positive, determined by radioimmunoassay or immunohistochemistry. Tumor size was categorized as <20, 20–40 and >40 in diameter (mm). Human epidermal growth factor receptor 2 (HER2) status, assessed by IHC/immunocytochemistry and confirmed by fluorescence *in situ* hybridization analysis if protein levels from IHC/immunocytochemistry showed 2+ or 3+, was recorded in the register as positive or negative. Lymph node involvement status was dichotomized (No/Yes). Registry information was essentially complete (98%) for tumor size and lymph node status, but with more missing data for ER status (80% complete). Grade was available from 2004 onward, with 93% completeness. Controls were breast cancer-free participants recruited between 2010 and 2011 from Helsingborg and Stockholm in Sweden, a subset of the KARMA study. All participants had been genotyped on the iCOGS array in accordance with relevant guidelines as described previously^26^ and missing genotypes were imputed using 1 KG (phase I integrated variant set release (v3) in the National Center of Biotechnology Information build 37 [hg19] coordinates). Each participant gave informed consent and this study has been approved by the ethical review board at Karolinska Institutet.

Celiac-PRS profiles for each individual were generated by summing the number of celiac disease risk allele copies weighted by effect estimates reported on the GWAS study by Trynka *et al*. ^17^, using a scoring routine in the PLINK program (version 1.9b3x)^63^. We computed four celiac-PRS profiles based on subsets of independent (r^2^ > 0.2) celiac disease SNPs defined by different P-value thresholds [*P*_CD_ < 5E-08 (*n* = 199), *P*_CD_ < 1E-05 (*n* = 276), *P*_CD_ < 0.01 (*n* = 1,284), and *P*_CD_ < 0.05 (*n* = 3,803)].

Statistical analyses were performed in R (version 3.2.4). Unconditional logistic regressions were used to estimate ORs and corresponding 95% CI interval for association of celiac-PRS with overall, ER-negative, and ER positive breast cancer risk. PRS profiles were tested as both a continuous variable per standard deviation (per-SD) and a categorical variable defined by quartiles (based on PRS distribution in breast cancer controls), with the lowest quartile as the reference. We also investigated whether celiac-PRS differentially influences breast cancer tumor characteristics in a case-only study: ER status, lymph node involvement, and HER2-status were tested as binary outcomes using binomial logistic regressions; tumor grade and tumor size were modeled as categorical variables using multinomial logistic regressions (with the “nnet” R package).

### Enrichment analysis on top-overlapping SNPs

To aid in the biological interpretation of top (lowest P-values) overlapping SNPs between celiac disease and breast cancer risk overall, we performed SNP enrichment analysis using DEPICT (version1 rel194)^30^, an integrative tool that systematically prioritizes the most likely causal genes at associated loci and highlights enriched pathways based on a pre-computed probability of gene set membership across 14,461 reconstituted gene sets. Loci within base pairs 25,000,000–35,000,000 on chromosome 6 are excluded due to the heightened LD seen on this major histocompatibility region.

## Electronic supplementary material

Supplementary information
