# Supplementary material for: Common shared genetic variation behind decreased risk of breast cancer in celiac disease
Source: Sci Rep. 2017 Jul 19;7:5942. doi: 10.1038/s41598-017-06287-9 (PMC5517429; doi:10.1038/s41598-017-06287-9)
Supplement: Supplementary file 1 — Supplementary information [file 41598_2017_6287_MOESM1_ESM.pdf]

Common shared genetic variation behind decreased risk of breast cancer in celiac disease

Emilio Ugalde-Morales, MSc;[1]* Jingmei Li, PhD;[1,2] Keith Humphreys, PhD;[1] Jonas F Ludvigsson, PhD;[1, 3] Haomin Yang, MSc;[1] Per Hall, PhD;[1] Kamila Czene, PhD [1].

[1] Department of Medical Epidemiology and Biostatistics, Karolinska Institutet, Stockholm, Sweden.

[2] Human Genetics, Genome Institute of Singapore, Singapore 138672, Singapore

[3] Department of Pediatrics, Örebro University Hospital, Örebro, Sweden

SUPLEMENTARY FIGURES AND TABLES

Supplementary Figure 1. Primary pleiotropic SECA tests.

Supplementary Figure 2. DEPICT sensitivity analysis.

Supplementary Table 1. LDSC filter procedure (in number of SNPs).

Supplementary Table 2. SECA filter procedure (in number of SNPs).

Supplementary Table 3. Celiac-PRS comparison within tumor characteristics (case-only analysis).

Supplementary Table 4. GWAS summary statistics for the 52 ‘top’ overlapping SNPs.

Supplementary Table 5. Enriched gene sets from DEPICT analysis on 52 ‘top’ overlapping SNPs.

Supplementary Figure 1. Primary pleiotropic SECA tests. a) Genetic overlap: SNP subsets with significant excess of overlapping SNPs ($P_{BT} < 0.05$) are highlighted in bright red; permuted P-values are indicated for each primary test and for the subset with minimum overlap ($P_{BT\text{-}min}$). b) Genetic concordance or discordance: SNP subsets yielding negative correlation ($OR_{FT} < 1$) are in green, or in orange positive ($OR_{FT} > 1$); subsets with significant discordance are highlighted in dark green ($P_{FT} < 0.05$).

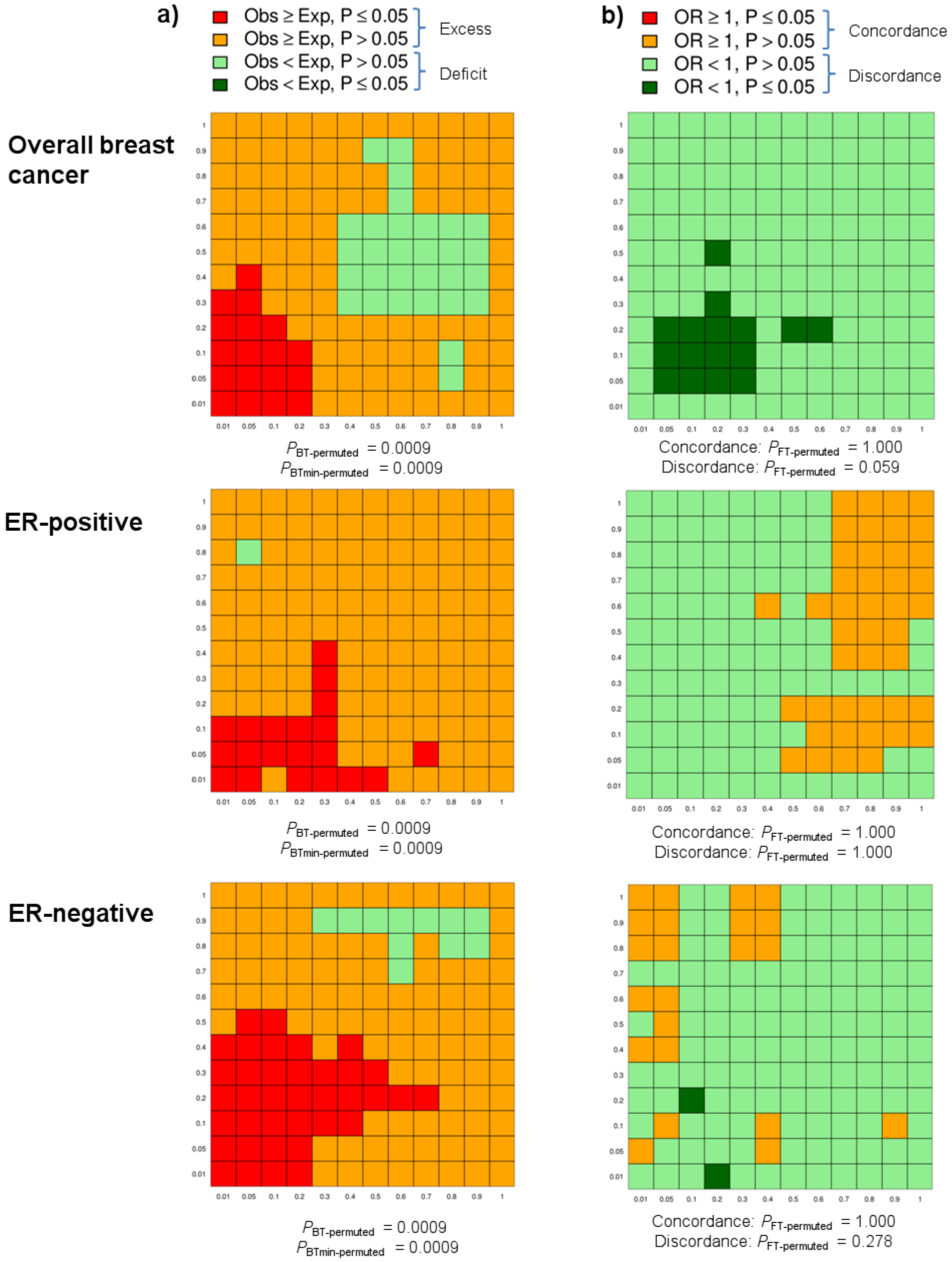

Supplementary Figure 2. DEPICT sensitivity analysis. Venn diagram comparing 15 prioritized genes (FDR_{DEPICT} <0.05) found using 52 'top' CD-SNPs (P_{CD} < 1E-05) in overlap with breast cancer at $P_{BC} \leq 0.05$ (left-sphere), and 19 genes (FDR_{DEPICT} <0.01) from 'sensitivity analysis' using 66 CD-SNPs (P_{CD} < 1E-05) with no association to breast cancer (P_{BC} > 0.05) (right-sphere).

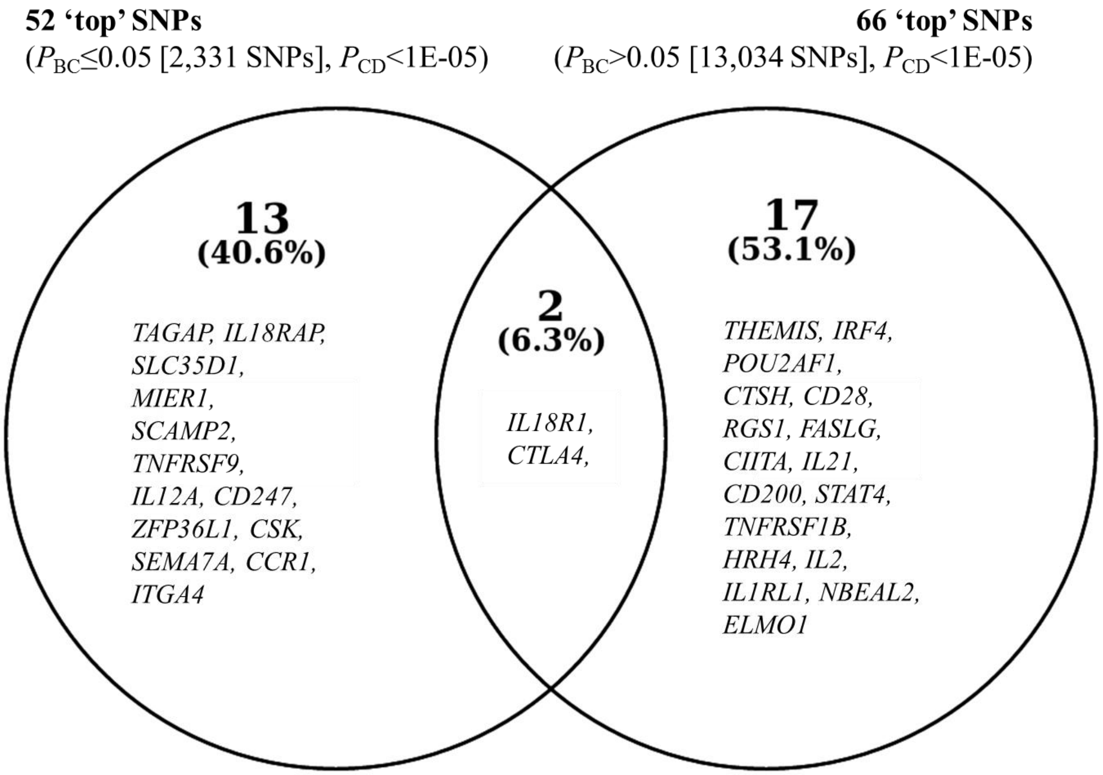

Supplementary Table 1. LDSC filter procedure (in number of SNPs).

	BC overall	ER-positive	ER-negative	Celiac disease
Input	173,301	173,301	173,301	129,618
Missing values	21,204	21,204	21,204	2,498
not SNP- or strand- ambiguous	0	0	0	377
P-value out of bounds	0	4	0	0
Duplicates	130	130	130	261
Not match –LD merge-alleles	97,909	97,909	97,909	80,816
Subtotal	54,058	54,054	54,058	45,666
‘LD merge-alleles’	53,358	53,354	53,358	45,500
Matching SNPs	45,500	45,500	45,500	-
With valid alleles (Total)	45,451	45,451	45,447	-

Number of (total) SNPs included in the LDSC analysis. SNP remaining after standard filtering procedure (subtotal), and SNPs for which pre-computed LD scores were available in the LDSC built-in data (‘LD merge-alleles’), are indicated.

Supplementary Table 2. SECA filter procedure (in number of SNPs).

	BC overall	ER-positive	ER-negative	CD
Input	173,301	173,301	173,301	129,618
Missing values	21,204	21,204	21,204	2,498
'Bad alleles'[§]	0	0	0	20
Duplicated in input	874	874	874	161
SNP ID's mismatch	0	0	0	7,578
After quality control	151,223	151,223	151,223	119,361
In overlap with CD	109,151	109,151	109,151	-
Independent SNPs	15,365	15,400	15,428	-

Number of independent SNPs used for SECA analyses after standard filtering of GWAS summary statistics files. [§], SNPs with mislabeled alleles were skipped, and for all datasets the first instance was used when marker was duplicated in summary statistics files.

Supplementary Table 3. Celiac-PRS comparison within tumor characteristics (case-only analysis).

Tumor characteristics	P_{CD}<5E-08 (199 SNPs)			P_{CD}<1E-05 (276 SNPs)			P_{CD}<0.01 (1,284 SNPs)			P_{CD}<0.05 (3,803 SNPs)		
	OR	95% CI	*P*-value	OR	95% CI	*P*-value	OR	95% CI	*P*-value	OR	95% CI	*P*-value
ER status	1.07	0.90,1.26	0.37	1.07	0.90,1.26	0.36	1.08	0.91,1.27	0.34	1.10	0.93,1.29	0.26
HER2 status	1.07	0.90,1.26	0.46	1.07	0.90,1.26	0.42	1.08	0.91,1.27	0.36	1.10	0.93,1.29	0.28
L. Node	1.01	0.95,1.07	0.82	1.01	0.94,1.07	0.85	1.00	0.94,1.07	0.90	1.01	0.95,1.07	0.79
Tumor grade	1.06	0.96,1.18	0.26	1.06	0.96,1.17	0.27	1.06	0.95,1.17	0.28	1.06	0.95,1.17	0.29
Tumor size	0.97	0.76,1.22	0.77	0.97	0.77,1.22	0.78	0.97	0.76,1.22	0.77	0.98	0.78,1.23	0.85

The profiles (and number of SNPs) are based on SNPs under four celiac P-value (P_{CD}) **t**hresholds. Number of individuals included in the analysis per tumor characteristic: ER status, positive (*n*=3,804) versus negative (*n*=695); HER2 status, positive (*n*=162) versus negative (*n*=1,056); Lymph node involvement, yes (*n*=1,392) versus no (*n*=3,002); Grade 1 as reference (*n*=618), grade 2 (*n*=1,631) and grade 3 (*n*=871); Tumor size (mm) <20 as reference (*n*=3,361), 20-40 (*n*=1,048) and >40 (*n*=76). ORs with 95% CIs are shown per 1-SD.

Supplementary Table 4. GWAS summary statistics for the 52 ‘top’ overlapping SNPs.

SNP	CHR	BP_b37	$BETA_{CD}$	P_{CD}	$BETA_{BC}$	P_{BC}
rs1015811	6	28448086	0.20899	4.93E-21	-0.03	0.0041
rs10484439	6	26309908	1.0156	3.07E-261	-0.07	0.00013
rs11216956	11	118575326	-0.14815	2.94E-11	-0.04	0.00017
rs114035824	6	31199164	-0.16688	2.20E-08	0.04	0.015
rs114129747	6	30836225	0.65739	6.11E-17	-0.08	0.05
rs114296351	6	29661456	-0.37819	1.92E-84	0.02	0.014
rs114461203	6	32272310	0.96024	2.36E-109	-0.04	0.0092
rs114502312	6	32968397	0.38254	6.47E-11	-0.11	0.0064
rs114570085	6	29829482	0.2567	6.96E-36	-0.04	0.00010
rs114716686	6	29816421	0.43634	4.08E-58	-0.04	0.0021
rs114762590	6	28916252	-0.23029	2.55E-33	0.05	1.30E-06
rs114885050	6	30117224	-0.21381	1.56E-09	-0.04	0.042
rs1150755	6	32038550	1.7572	9.88E-324	-0.03	0.012
rs115102354	3	46222037	-0.27231	4.57E-11	0.06	0.0036
rs115125931	6	30433090	0.34136	1.10E-11	-0.08	0.0041
rs115258774	6	30390199	-0.48551	3.98E-142	0.04	7.10E-05
rs115347502	6	30933864	-0.56335	2.00E-180	0.02	0.011
rs115560311	6	30740515	-0.4219	5.99E-48	0.03	0.028
rs115820457	6	30389347	-0.42098	1.00E-17	0.05	0.035
rs115959109	6	30505000	0.55513	2.45E-29	0.07	0.0045
rs116296367	6	32196805	-0.36615	3.82E-16	-0.09	0.00018
rs116714959	6	30038383	-0.27879	2.89E-07	-0.09	0.00069
rs116793141	6	31448625	-0.29062	1.50E-34	0.04	0.00078
rs11680095	2	181825956	-0.08526	4.72E-06	0.02	0.044
rs116925127	6	29292030	0.33687	1.65E-08	-0.06	0.026
rs11847049	14	69259406	-0.12045	4.74E-08	0.03	0.0044
rs11903660	2	204711588	0.17479	8.50E-06	0.04	0.042
rs12615783	2	61440947	0.10225	1.02E-07	0.02	0.03
rs12661782	6	28466442	0.19177	2.28E-08	-0.06	0.00013
rs12713433	2	61307982	-0.10526	3.56E-06	-0.05	0.00059
rs140924029	6	31275231	0.37295	1.04E-08	0.07	0.039
rs1570760	6	33622933	-0.14514	2.12E-10	0.02	0.036
rs1972346	10	81067480	0.11968	3.56E-10	0.03	0.011
rs2071542	6	32811645	0.59131	1.16E-26	-0.06	0.0072
rs210134	6	33540209	0.23902	6.67E-34	-0.03	0.011
rs210203	6	33511229	-0.17889	5.12E-20	-0.02	0.037
rs225132	1	8095500	-0.11065	4.22E-06	0.04	0.00029
rs2256974	6	31555392	-0.51517	4.06E-79	0.03	0.0073
rs2296328	6	33651277	0.29788	2.50E-44	-0.03	0.014
rs2499714	6	34072215	0.18897	6.08E-10	0.04	0.042
rs2755244	1	67484470	-0.09497	9.86E-06	0.02	0.025
rs2847266	18	12773338	-0.090754	9.08E-06	0.03	0.0066
rs3819714	6	32804217	-0.18248	3.13E-19	0.03	0.012
rs4151670	6	31915532	-0.67846	7.70E-20	0.08	0.0058
rs4886410	15	75065644	0.11154	1.16E-08	0.02	0.037
rs6518350	21	45621817	0.11575	1.14E-06	0.04	0.0097

SNP	CHR	BP_b37	$BETA_{CD}$	P_{CD}	$BETA_{BC}$	P_{BC}
rs76830965	3	159637678	0.30748	2.57E-27	-0.03	0.041
rs78756788	2	103050569	-0.19557	6.66E-06	0.11	0.0013
rs79715597	6	159366373	-0.20701	7.95E-08	-0.06	0.0033
rs864537	1	167411384	0.084905	5.95E-06	0.03	0.002
rs9347286	6	159515360	-0.15269	4.72E-08	0.04	0.0028
rs9865818	3	188072513	0.23674	1.72E-35	-0.02	0.046

CHR, chromosome; BP_b37, base-pare position on the b37 genome assembly.

Supplementary Table 5. Enriched gene sets from DEPICT analysis on 52 ‘top’ overlapping SNPs.

Original gene set ID	Original gene set description	Nominal *P*-value
GO:0006917	induction of apoptosis	1.10E-09§
GO:0012502	induction of programmed cell death	1.30E-08
GO:0048534	hemopoietic or lymphoid organ development*	6.87E-08
MP:0000322	increased granulocyte number*	1.41E-07
GO:0030097	hemopoiesis*	3.39E-07
ENSG00000182481	KPNA2 PPI subnetwork	3.86E-07
ENSG00000215769	ENSG00000215769 PPI subnetwork	3.86E-07
GO:0030099	myeloid cell differentiation*	9.25E-07
ENSG00000135341	MAP3K7 PPI subnetwork	1.77E-06
MP:0005016	decreased lymphocyte cell number	2.14E-06
MP:0008214	increased immature B cell number*	3.57E-06
MP:0000221	decreased leukocyte cell number	7.50E-06
GO:0002521	leukocyte differentiation*	1.55E-05
ENSG00000112062	MAPK14 PPI subnetwork	1.83E-05
ENSG00000065559	MAP2K4 PPI subnetwork	2.33E-05
GO:0002573	myeloid leukocyte differentiation	2.36E-05
MP:0009763	increased sensitivity to induced morbidity/mortality	2.82E-05
MP:0000333	decreased bone marrow cell number	2.89E-05
MP:0000240	extramedullary hematopoiesis*	3.05E-05
ENSG00000101966	XIAP PPI subnetwork	3.89E-05
ENSG00000115145	STAM2 PPI subnetwork	4.99E-05

Hits with FDR adjusted P-value lower than 0.05. *, hits also found significant (FDR < 0.01) in the sensitivity analysis (66 CD-SNPs, P_{BC} > 0.05) were considered as unreliable findings. §, FDR<0.01.
